# Great Britain

**Published:** 1896-08

**Authors:** 


*Great Britain.* Through the passage of the "Diseases of Animals" act, the regulation in force for several years becomes permanent, that from January 1st, 1897, all animals from foreign countries will have to be slaughtered at the point of landing. Animals imported for breeding or other special purposes will be subjected to quarantine regulations.

